# Endovascular therapy for erectile dysfunction: insights from more than 1000 treatments

**DOI:** 10.1186/s42155-026-00666-y

**Published:** 2026-02-28

**Authors:** Nicolas Diehm, Patrick Duy Dang Do, Madlen Röllig, Dominik Müller, Sebastian Sixt, Michael Glenck, Andreas Gutwein, Tamara Walser, Chiara Brechbühl, Martin Christian Schumacher, Dai-Do Do, Hanno Hoppe

**Affiliations:** 1 Vascular Institute Central Switzerland, Aarenaustrasse 2B, Aarau, 5000 Switzerland; 2 Faculty of Medicine, University of Bern, Murtenstrasse 11, Bern, 3008 Switzerland; 3 University of Applied Sciences Furtwangen, Jakob-Kienzle-Strasse 17, Villingen-Schwenningen, 78054 Germany; 4 Department of Vascular Surgery, Inselspital University Hospital Bern, Freiburgstrasse 20, Bern, 3008 Switzerland; 5 Vascular Center Biel, Bahnhofstrasse 14, 2502 Biel, Switzerland; 6 Microtherapy Center Bern, Lindenhofgruppe, Bremgartenstrasse 117, Bern, 3012 Switzerland; 7 Hirslanden Clinic Aarau, Urology Center, Aarau, Schänisweg, 5001 Switzerland; 8 Campus Stiftung Lindenhof Bern, Swiss Institute for Translational and Entrepreneurial Medicine, Hochfeldstrasse 41, Bern, 3012 Switzerland; 9 Department of Diagnostic, Interventional and Pediatric Radiology (DIPR), Inselspital, Bern University Hospital, University of Bern, Rosenbühlgasse 27, Bern, 3010 Switzerland; 10 Department of Health Sciences and Medicine, University of Lucerne, Frohburgstrasse 3, Postfach 4466, Lucerne, 6002 Switzerland

**Keywords:** Erectile dysfunction, Endovascular therapy, Arterial, Venous leak, Embolization

## Abstract

**Background:**

To report effectiveness and patient-reported satisfaction after endovascular treatment of vasculogenic erectile dysfunction (ED) in a large registry cohort.

**Methods:**

Erectile function was assessed using the International Index of Erectile Function (IIEF) questionnaires. Endovascular treatments included revascularization of arterial obstruction and/or venous leak embolization. Safety endpoints included the absence of device- or procedure-related major adverse events. Patient satisfaction was measured at 6 weeks using the Patient Global Impression of Improvement (PGI-I) questionnaire.

**Results:**

According to the SwissPower registry, 1032 endovascular procedures were performed on 776 patients, thereof 524 patients received at least one arterial revascularization, and 188 men underwent at least one venous embolization. Of note, 64 patients underwent both venous plus arterial procedures during the study period. Among patients with available follow-up IIEF-6 data, a ≥ 4-point improvement was observed in 175/402 men (44%) after arterial revascularization at 30 months, in 62/140 men (44%) after venous leak embolization at 9 months, and in 28/54 men (52%) after combined arterial plus venous interventions at 21 months. According to PGI-I, patients reported improved erectile function after arterial revascularization in 61% (226/369), venous leak embolization in 72% (102/142), and combined arterial plus venous interventions in 68% (38/56). Overall, 400/554 men (72.2%) reported that they would undergo the procedure again: 67.4% after arterial intervention, 78.7% after venous embolization, and 87.5% after combined arterial plus venous interventions.

**Conclusions:**

In this large registry cohort, endovascular treatment for vasculogenic erectile dysfunction was associated with clinically meaningful improvement of erectile function in the majority of patients.

**Graphical Abstract:**

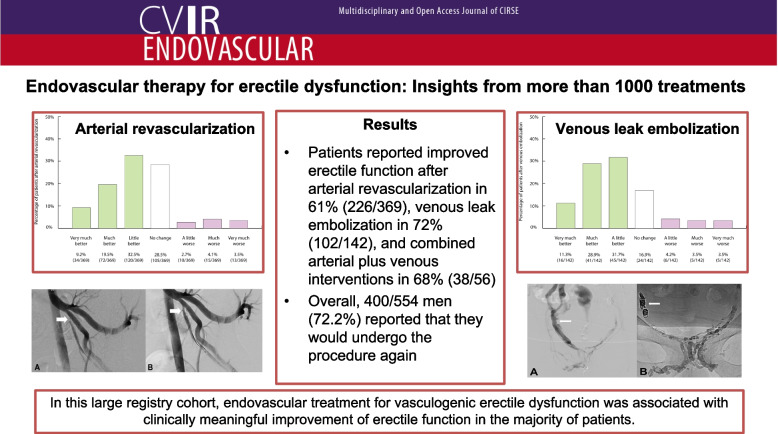

## Introduction

Erectile dysfunction (ED) is defined as the recurrent inability to achieve and maintain satisfactory erections for sexual intercourse and is known to be associated with a notable impairment of quality of life and personal relationships [1].

ED is a prevalent condition affecting an estimated 30–50% of men over the age of 40, with the incidence increasing significantly with age and comorbidities such as cigarette smoking, diabetes mellitus, arterial hypertension, and cardiovascular disease [2].

Arterial obstruction of the pelvic arteries and venous leak, alone or in combination, plays a critical role in the pathophysiology of vascular ED. Vascular pathologies account for the majority of cases of severe ED that do not respond to vasoactive agents such as phosphodiesterase-5 inhibitors (PDE5is) or intracavernous prostaglandins [3].

Conventional therapies, including PDE5is, intracavernosal injections, and vacuum erection devices, have demonstrated varying degrees of success [4]. However, these approaches primarily address symptoms rather than the underlying vascular pathology. In addition, up to one in two patients taking PDE5is report erections that are inadequate for sexual intercourse or report drug-related side effects.

Recently, advances in diagnostic imaging and endovascular techniques have paved the way for novel endovascular therapies to restore penile perfusion and occlude venous leak. This growing body of evidence supports the clinical success of endovascular therapy in selected patients [3, 5, 6]. In this study, our data and proceedings on more than 1000 endovascular ED procedures are reported.

## Patients and methods

For this prospectively maintained multicenter study, patients with severe ED who were enrolled in the SwissPower registry between March 2016 and October 2024 were included. This registry is an ethics-approved, prospective registry used for investigating endovascular treatment of vasculogenic ED (Ethics approval: EKNZ 2018–00408). All study procedures adhered to the principles of the Declaration of Helsinki and Good Clinical Practice guidelines. Written informed consent was obtained from all participants who retained the right to withdraw their data at any time. All the patients included in the present study have data in the SwissPower registry. Thus, patients were partly included in previous studies [7–11].

### Patients

Patients were treated at two vascular centers, either presenting on their own initiative or through referrals from urologists, general practitioners, or cardiologists. Endovascular treatment was performed in patients with confirmed arterial and/or venous etiology in whom the maximum dose of PDE-5-i failed to provide erections sufficient for satisfactory intercourse, was contraindicated, or resulted in adverse effects that precluded their continued use. In patients with combined arteriogenic and venogenic ED, arterial revascularization was performed as a priority [10]. All patients had undergone prior urological evaluation, during which urological causes of ED were either excluded or appropriately treated before vascular assessment.

### Penile duplex sonography and computed tomography

**Fig. 1:**
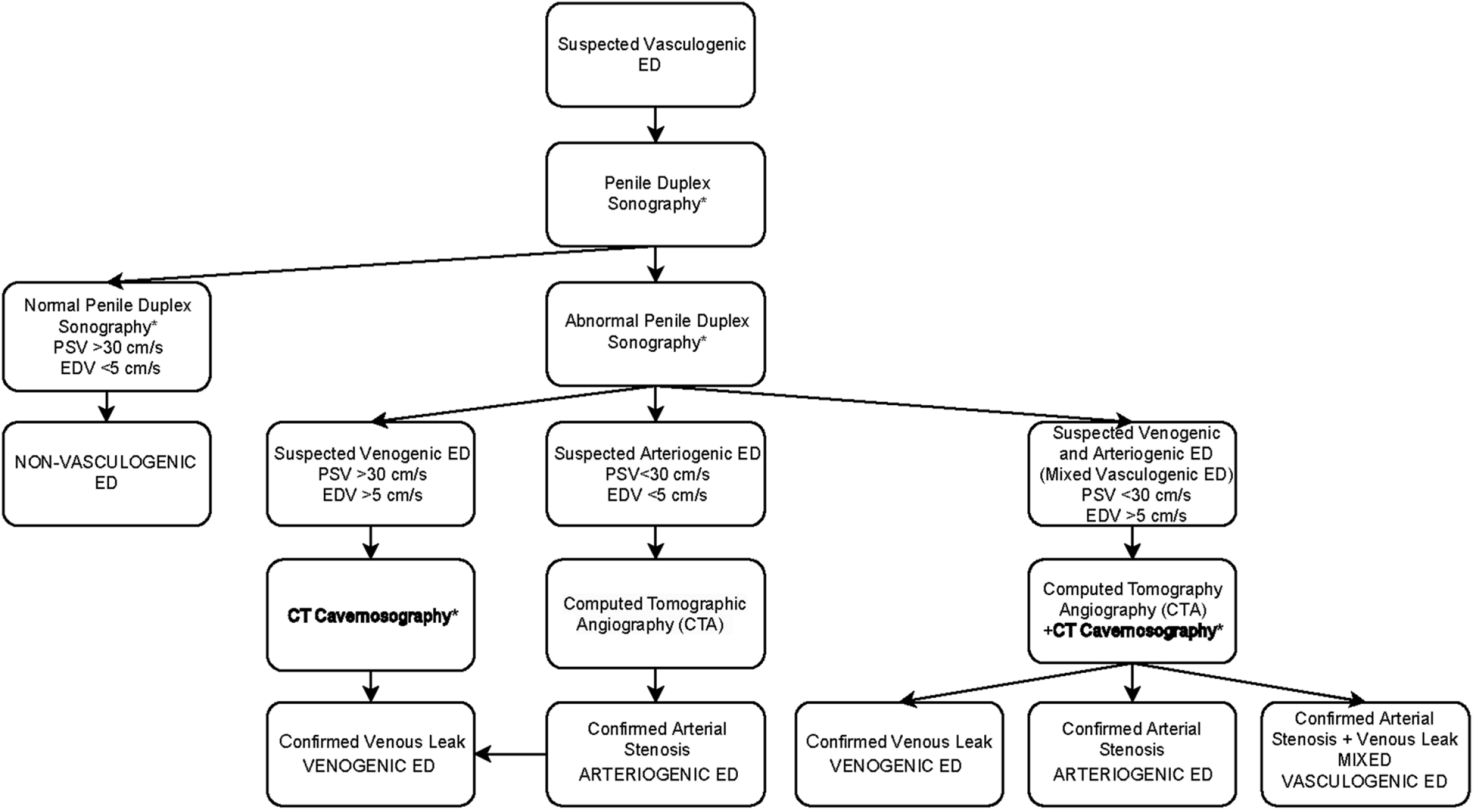
Suggested diagnostic management algorithm for patients with vasculogenic erectile dysfunction, including CT cavernosography (bold), for diagnosing patients with venogenic erectile dysfunction (ED – erectile dysfunction, PSV – peak systolic velocity, EDV – end diastolic velocity, *— postintracavernosal injection of alprostadil). Figure unmodified from Hoppe et al. (CVIR Endovasc). 2023 Nov 17;6(1):56. https://doi.org/10.1186/s42155-023-00403-9. http://creativecommons.org/licenses/by/4.0/

Preinterventional patient work-up, including penile duplex sonography and computed tomography cavernosography, was performed as previously described [8, 10, 12–15]. The work-up algorithm is demonstrated in Fig. 1.

### Arterial revascularization

**Fig. 2:**
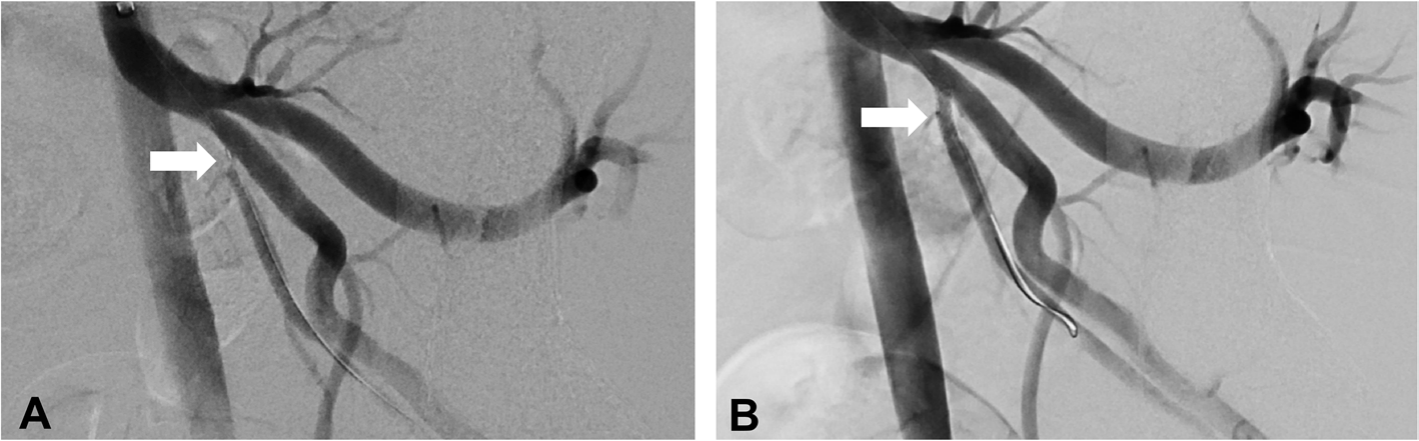
Arterial revascularization procedure: 54-year-old male patient with arteriogenic ED. **A** Arteriogram demonstrating high grade stenosis of the proximal left internal pudendal artery (arrow). **B** Revascularization was performed placing a 2.75 × 13 mm drug-eluting stent (arrow)

Currently, our treatment algorithm prioritizes primary endovascular revascularization of arteries relevant to erectile function in patients with combined arteriogenic and venogenic ED. If erectile function remained insufficient despite technically successful arterial revascularization, secondary venous leak embolization was performed. Arterial revascularization was performed as described previously (Fig. 2) [8].

Patients were re-evaluated within 6 weeks postintervention. If the clinical response was inadequate, penile duplex ultrasound was repeated to assess hemodynamics, diagnose potential arterial restenosis or occlusion, and determine whether a venous leak was present. Patients who continued to experience insufficient erectile function and who demonstrated no response to PDE5is, despite successful unilateral or bilateral revascularization and positive diagnostic findings of venous leak, were subsequently scheduled for venous leak embolization.

### Venous leak embolization

**Fig. 3:**
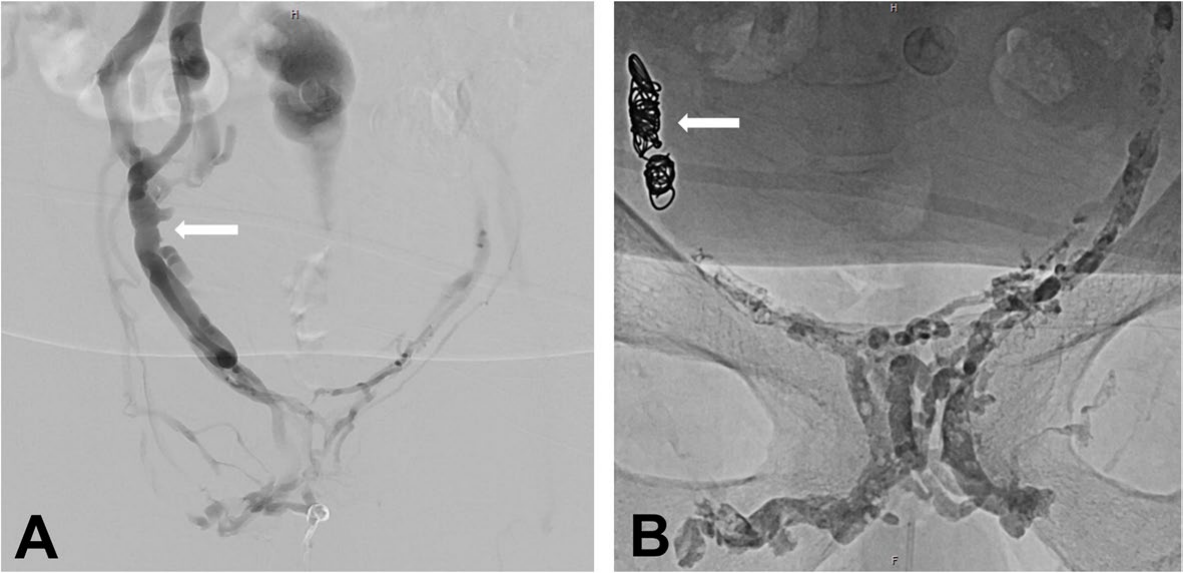
Venous leak embolization procedure: 68-year-old patient with venogenic ED. **A** Venogram demonstrating venous leak via internal pudendal veins and periprostatic venous plexus and fast outflow towards iliac veins via the right internal pudendal vein (arrow). **B **After protective coiling of the right internal pudendal vein (arrow), embolization with glue was performed

Venous leak embolization via penile or femoral venous access was performed as previously described (Fig. 3) [9, 11, 16–18].

### Medical therapy

During endovascular interventions, patients received an initial bolus of 5000 IU of heparin at the time of introducer sheath placement. Immediately following stent deployment, an oral loading dose of 300 mg of clopidogrel was administered. After completion of the diagnostic and therapeutic procedures, patients with atherosclerotic ED were prescribed acetylsalicylic acid (100 mg/day). Furthermore, when indicated, patients were started on lipid-lowering therapy (e.g., statins).

In cases of stent implantation, antiplatelet therapy consisted of 75 mg of clopidogrel once daily after the initial loading dose, with dual antiplatelet therapy continued for 6 months, followed by lifelong aspirin (100 mg/day). After venous embolization, patients received a 7-day course of ciprofloxacin (500 mg twice daily), ibuprofen (600 mg up to three times daily if needed), and pantoprazole (40 mg once daily). Additional oral analgesics were provided as needed. Following both endovascular interventions, tadalafil (5 mg daily with up to 20 mg on demand) was also prescribed for 3 weeks.

### Outcome assessment and study endpoints

Outcome assessment was performed per patient for the three subgroups “arterial revascularization,” “venous embolization,” and “arterial revascularization plus venous embolization.” Procedural assessment was analyzed per case.

Technical success for arterial revascularization was defined as the ability to obtain vascular access, complete the intended intervention, and achieve residual stenosis of less than 30%. Technical success for venous embolization was defined as complete and satisfactory embolization of the targeted vein. A visual analog scale (range 1–10) was used to quantify pain immediately after application of the embolic agent.

The primary safety endpoint was the absence of device- or procedure-related mortality or major adverse events, including target vessel necrosis, gangrene, symptomatic deep vein thrombosis, or pulmonary embolism. Periprocedural complications were classified using the CIRSE grading system [19].

To evaluate clinical efficacy, all participants completed the International Index of Erectile Function-15 (IIEF-15) questionnaire at baseline and again 6 weeks after the intervention. This validated tool assesses erectile function, orgasmic function, sexual desire, and overall satisfaction. The effect of treatment on erectile function was analyzed using the IIEF-6 subdomain.

A minimum clinically important difference (MCID) was defined as an increase of ≥ 4 points in the IIEF-6 score, which is consistent with previously established criteria in endovascular ED research [20]. A procedure was considered clinically feasible if at least 50% of treated individuals reached this improvement threshold.

In addition, patient responses to IIEF-15 questions 3 (ability to achieve penetration) and 4 (maintenance of sufficient erection during intercourse) were examined individually. Finally, the Patient Global Impression of Improvement (PGI-I) scale was administered at the 6-week follow-up, where patients rated their overall change on a 7-point scale ranging from “very much better” to “very much worse” [21]. In addition, patients were asked whether they would opt to undergo the treatment again.

### Statistical analysis

Continuous variables are presented as mean ± standard deviation (SD), and categorical variables are presented as absolute numbers and percentages. Group differences in continuous variables were compared using Student’s t test, the Mann–Whitney U test, or the Wilcoxon signed-rank test. Statistical significance was set at a two-sided value of *p* < 0.05. All analyses were conducted using XLSTAT version 2015.6.01.24026 (Addinsoft SARL).

## Results

**Fig. 4:**
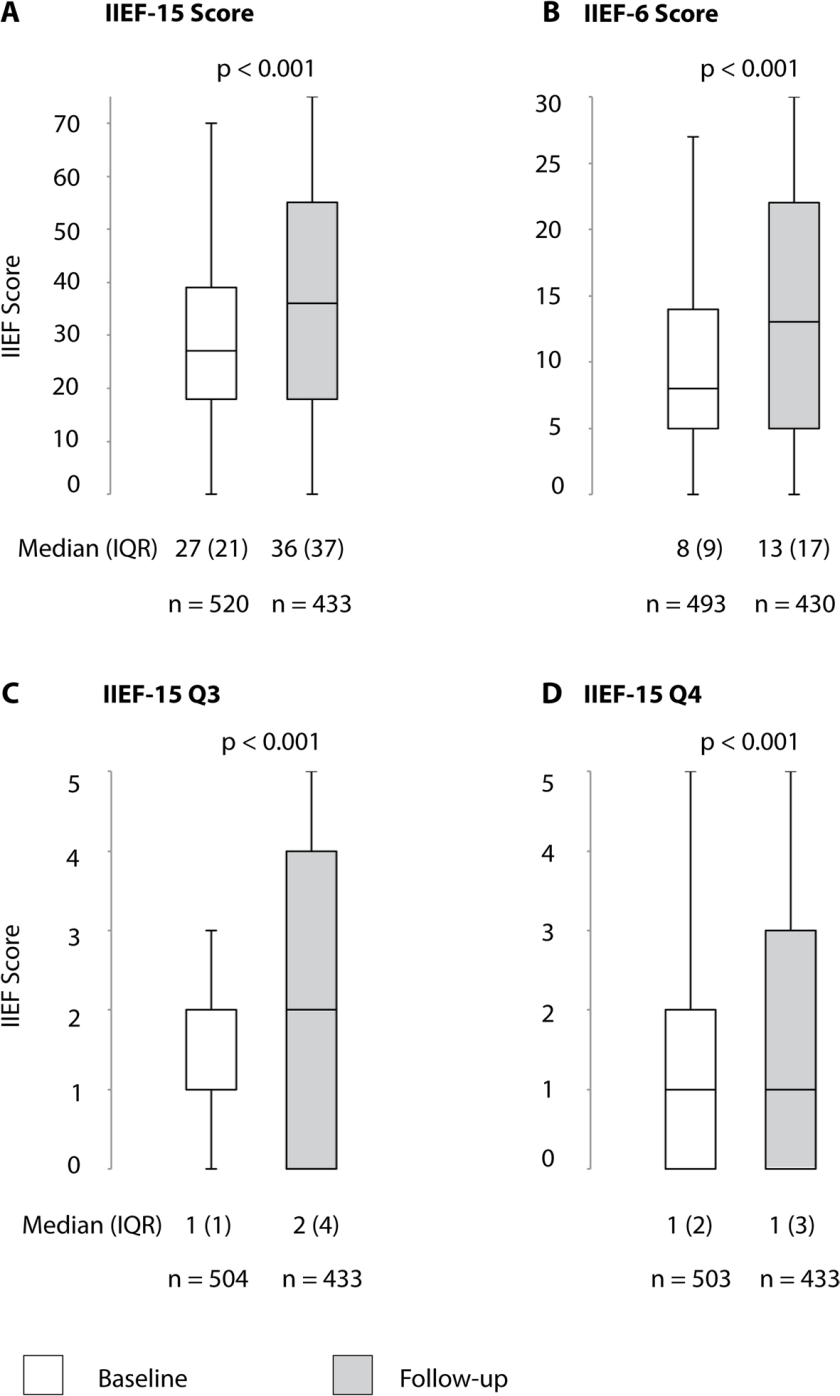
Changes in erectile function after arterial revascularization for arterial ED. IIEF 15 = International Index of Erectile Function (Questions 1–15). IIEF 6 = Domain questionnaire for erectile function (IIEF-15 questions 1, 2, 3, 4, 5, 15). IIEF-15 Q3 = Question 3 of the IIEF questionnaire (Ability to penetrate). IIEF-15 Q4 = Question 4 of the IIEF questionnaire (Ability to maintain erection)

**Fig. 5:**
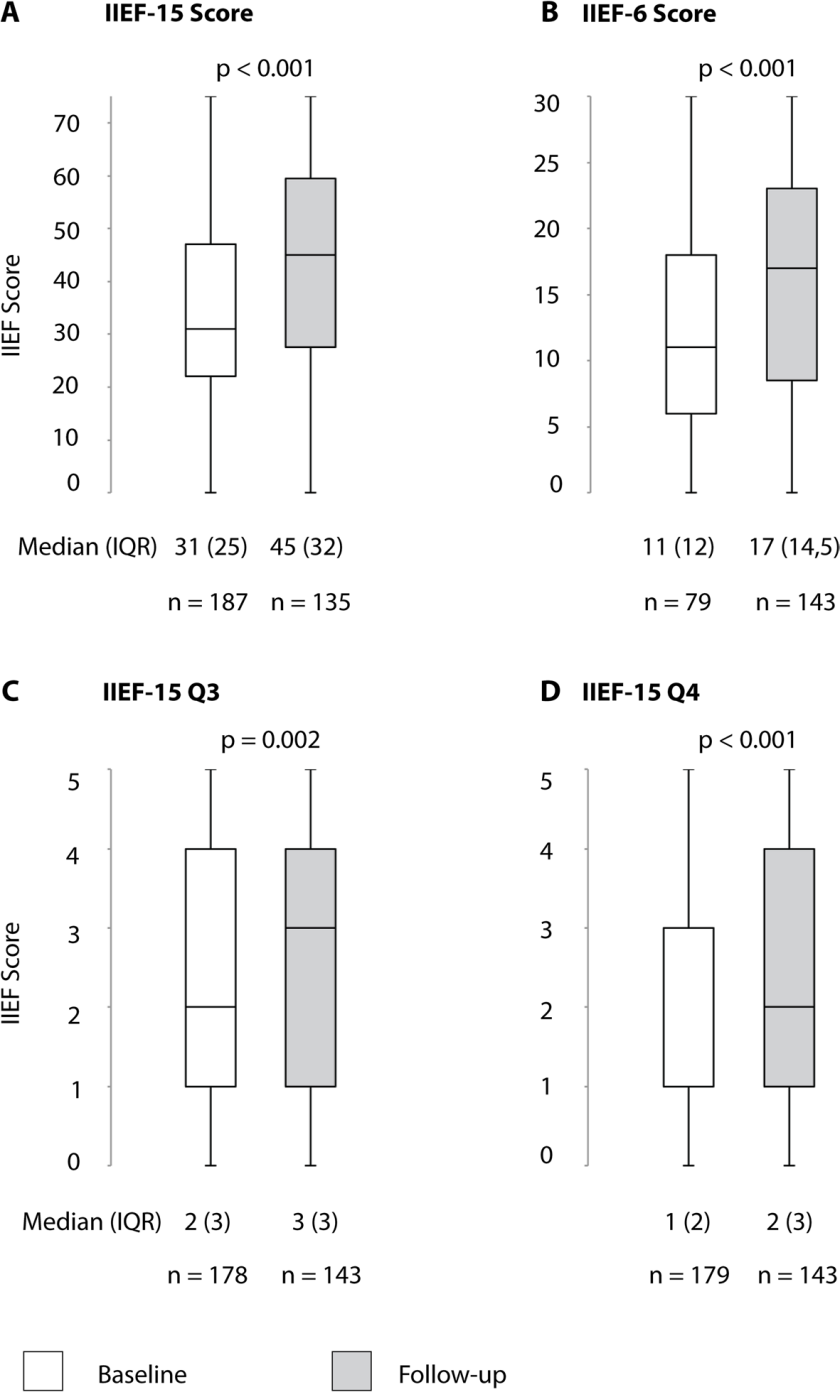
Changes in erectile function after venous embolization for venogenic ED. IIEF 15 = International Index of Erectile Function (Questions 1–15). IIEF 6 = Domain questionnaire for erectile function (IIEF-15 questions 1, 2, 3, 4, 5, 15). IIEF-15 Q3 = Question 3 of the IIEF questionnaire (Ability to penetrate). IIEF-15 Q4 = Question 4 of the IIEF questionnaire (Ability to maintain erection)

**Fig. 6:**
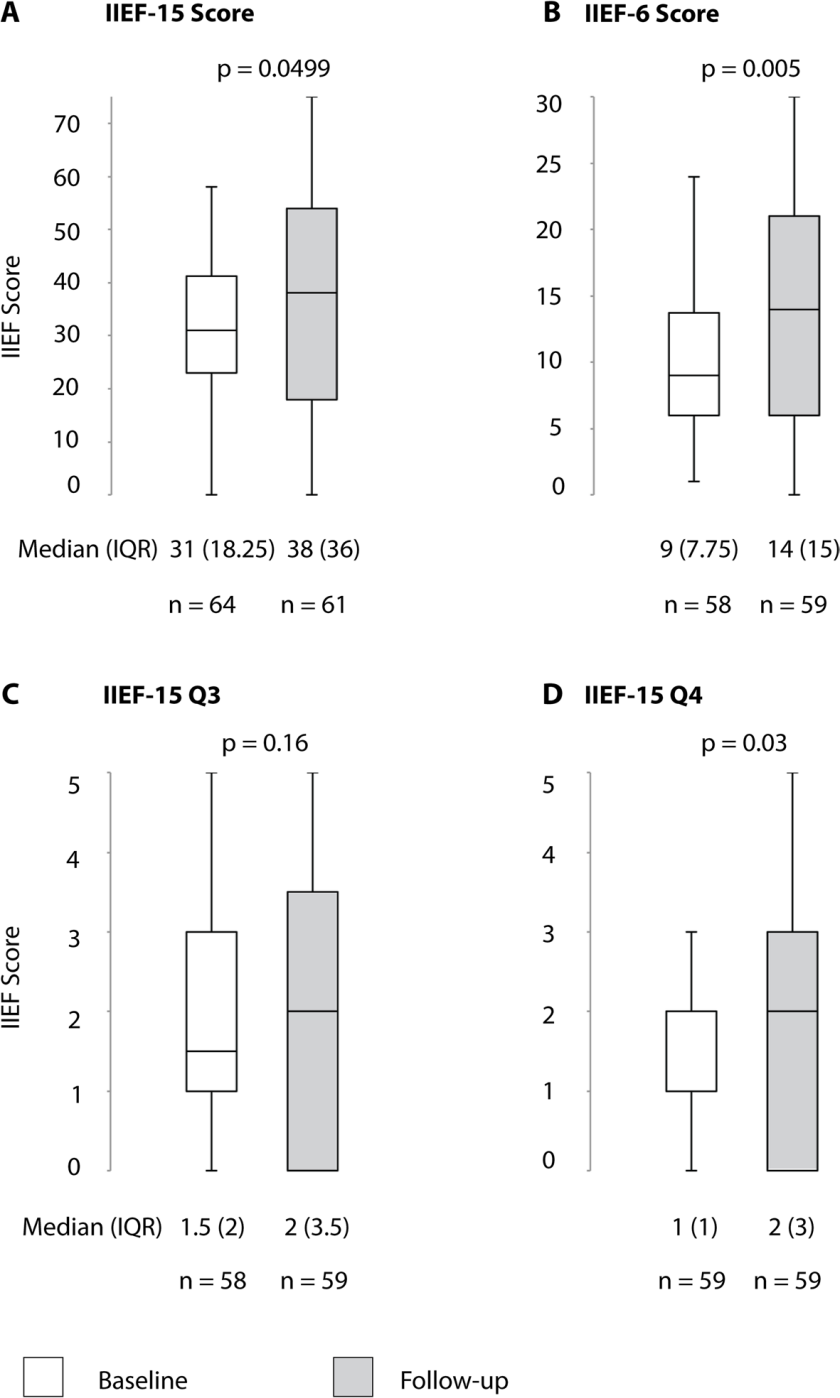
Changes in erectile function after arterial revascularization plus venous embolization. IIEF 15 = International Index of Erectile Function (Questions 1–15). IIEF 6 = Domain questionnaire for erectile function (IIEF-15 questions 1, 2, 3, 4, 5, 15). IIEF-15 Q3 = Question 3 of the IIEF questionnaire (Ability to penetrate). IIEF-15 Q4 = Question 4 of the IIEF questionnaire (Ability to maintain erection)

**Fig. 7:**
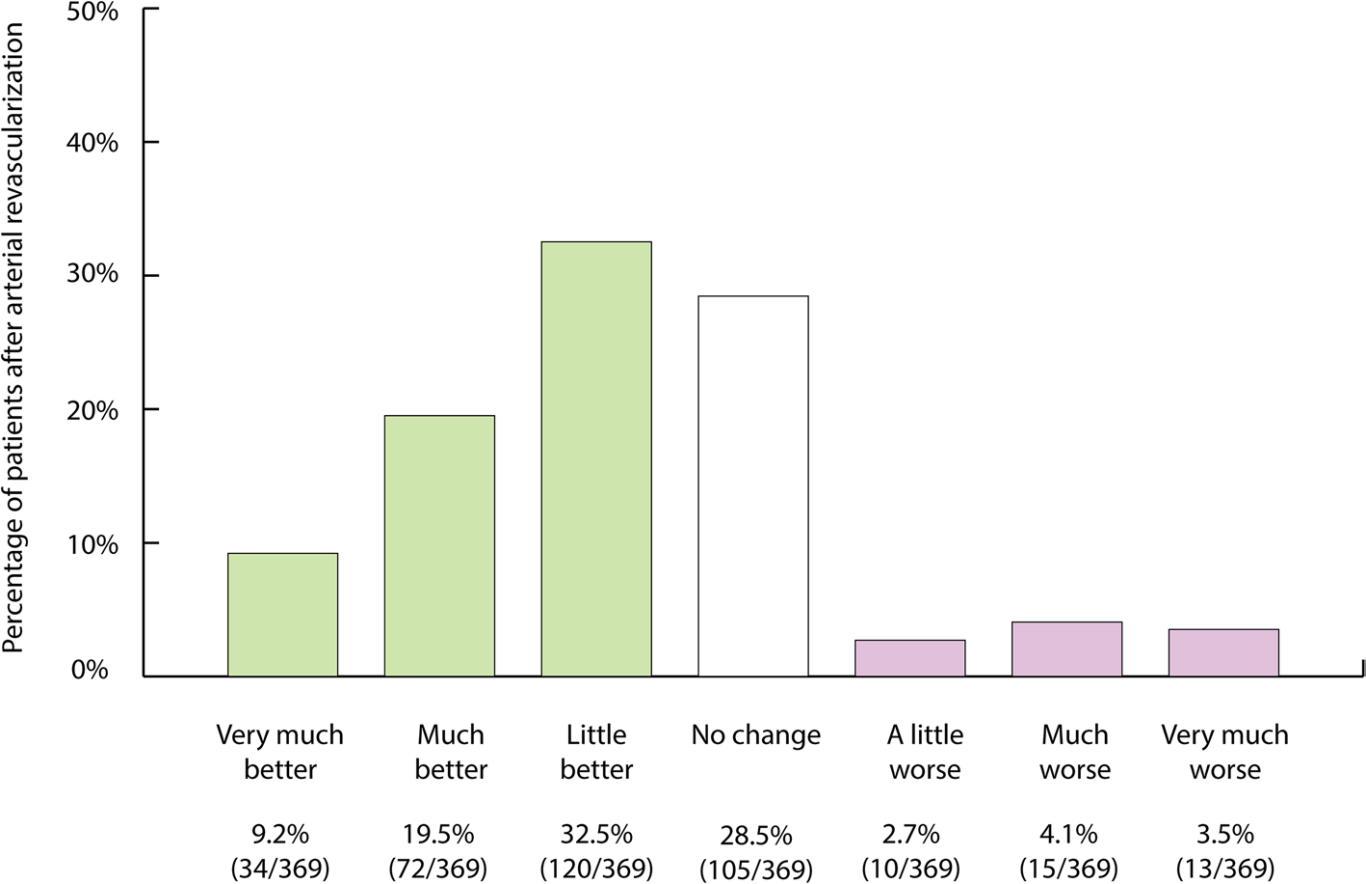
Assessment of patient outcomes after arterial revascularization on the basis of patient global impression of improvement [19]

**Fig. 8:**
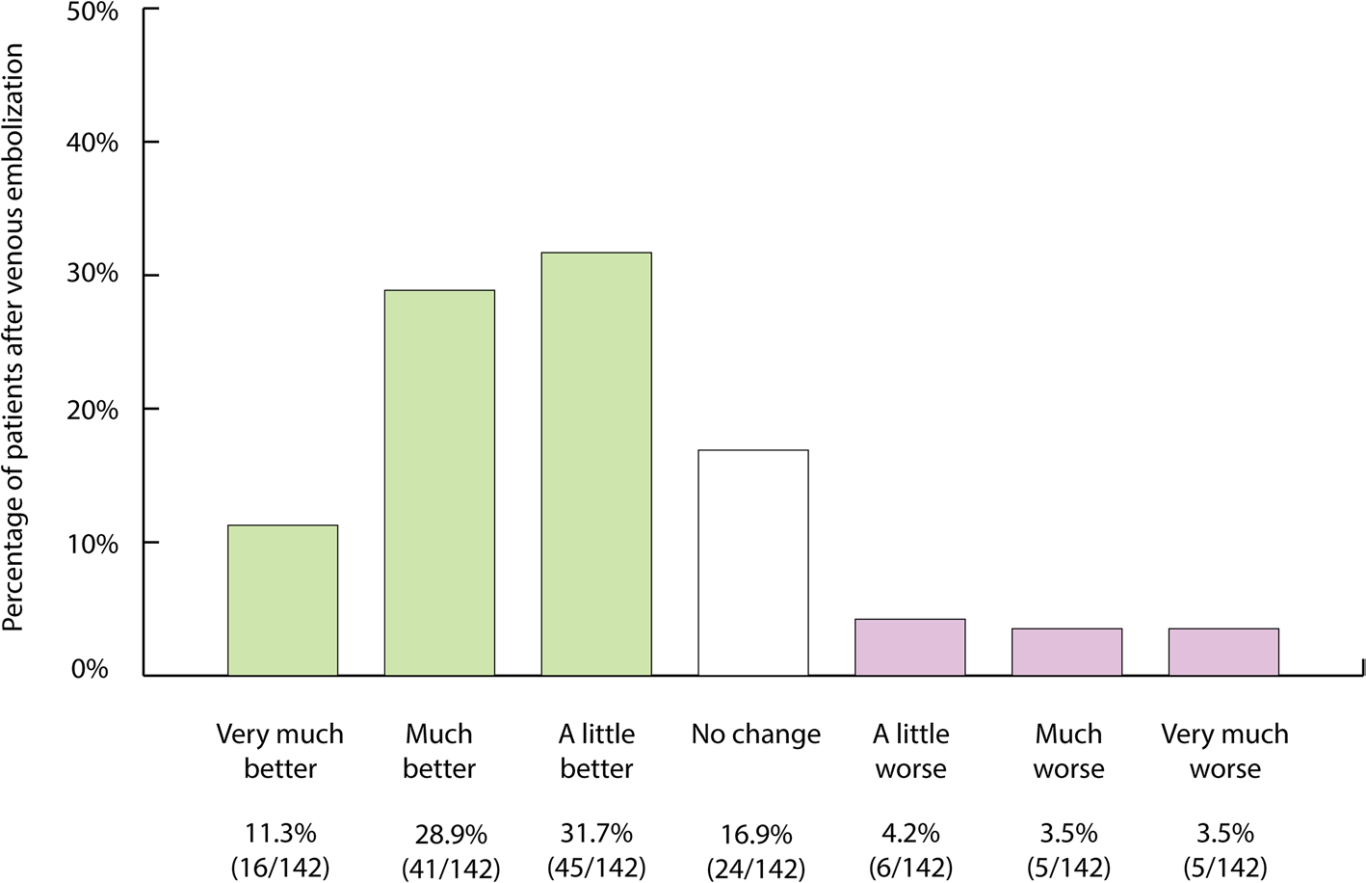
Assessment of patient outcomes after venous embolization on the basis of patient global impression of improvement [19]

**Fig. 9:**
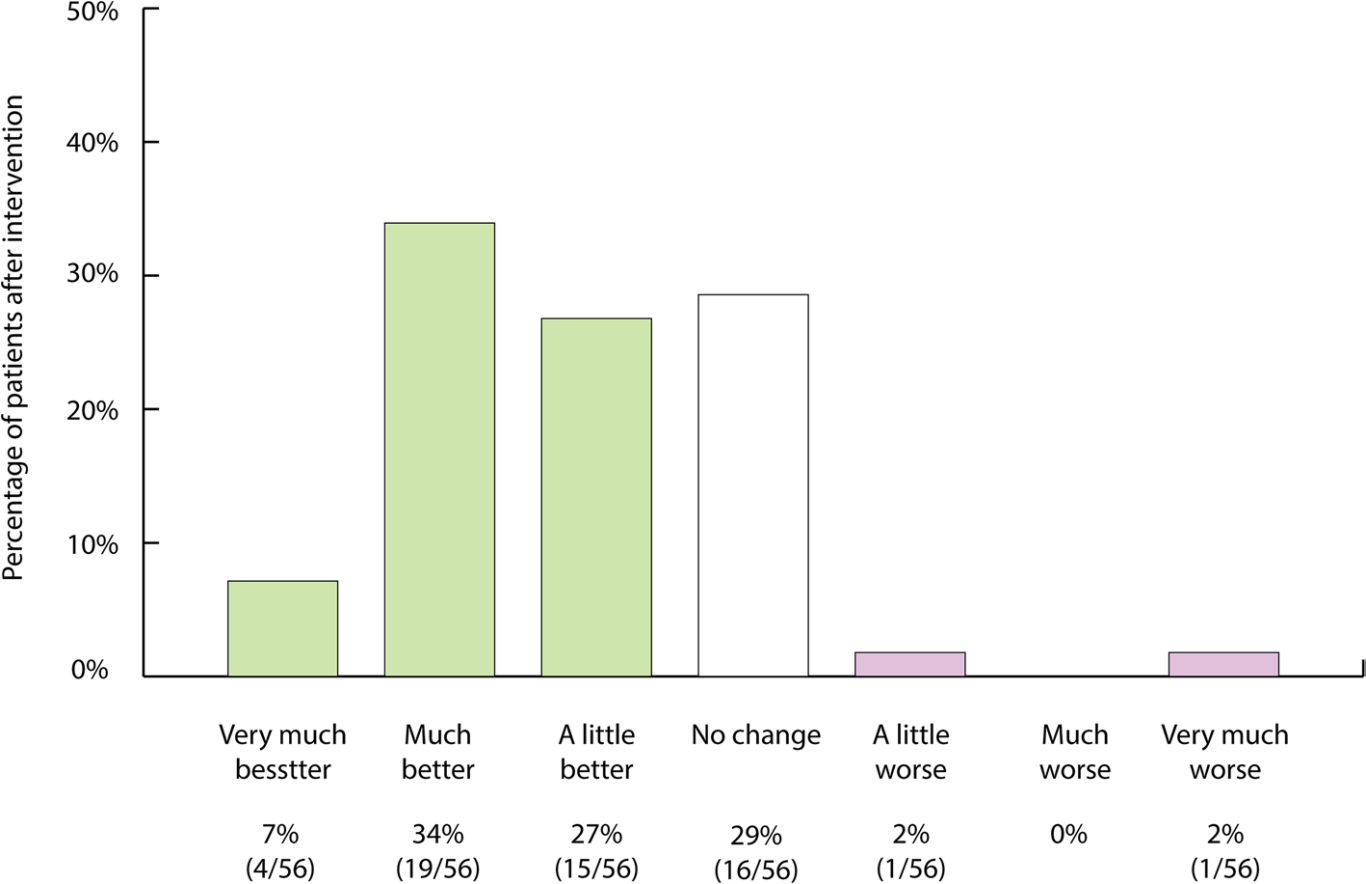
Assessment of patient outcomes after arterial revascularization plus venous embolization for mixed arterio- and venogenic ED based on patient global impression of improvement [19]

According to the SwissPower registry, 1032 endovascular procedures were performed on 776 patients, thereof 524 patients received at least one arterial revascularization and 188 men underwent at least one venous embolization (Tables 1, 2, 3, 4, and 5). Of note, 64 patients underwent both arterial revascularization plus venous embolization during the study period. Follow-up IIEF-15 questionnaires were evaluated venous in 629 (81.1%) of 779 patients with arteriogenic and/or venogenic ED (Table 6). Improvements in both IIEF-15 and IIEF-6 scores were statistically significant following either arterial or venous interventions (Figs. 4 and 5) as well as after arterial plus venous interventions for combined ED (Fig. 6). Figures 7, 8, and 9 provide an overview of patient satisfaction with treatment efficacy. A total of 400 out of 554 (72.2%) men stated that they would undergo the procedure again.

**Table 1:** Patient demographic characteristics, risk factors, and comorbidities of patients with arteriogenic erectile dysfunction before treatment

Patient characteristics	
Age, years	60 (SD10)
Nicotine abuse, active	143/339 (42.2%)
Nicotine abuse, former	74/339 (21.8%)
Diabetes mellitus	114/311 (36.7%)
Arterial hypertension	289/398 (72.6%)
Hyperlipidemia	274/388 (70.6%)
Hyperlipoproteinemia a	210/411 (51.1%)
Coronary artery disease	108/309 (35.0%)
Peripheral arterial occlusive disease	61/288 (21.2%)
Cerebrovascular disease	17/262 (6.5%)
Neurological disease(s), nonvascular	13/261 (5.0%)
Prior prostate surgery	29/260 (11.2%)
Chronic prostatitis	19/262 (7.3%)
Renal insufficiency ^a^	22/457 (4.8%)
Alcohol abuse, active	9/258 (3.5%)
Alcohol abuse, former	3/258 (1.2%)
Drug abuse, active	2/252 (0.8%)
Drug abuse, former	2/252 (0.8%)
Hormonal disorder	3/252 (1.2%)
Antiandrogens	1/522 (0.2%)
Antipsychotics	4/517 (0.8%)
Antidepressants	23/506 (4.5%)
Antihistamines	5/516 (1.0%)
Antidiabetics	14/99 (14.1%)
Antihypertensives	136/385 (35.3%)
Aspirin	113/379 (29.8%)
Oral anticoagulation	27/489 (5.5%)
Clopidogrel	17/506 (3.4%)
Statin	34/82 (41.5%)

Mean value (standard deviation (SD)) or number of patients (n/N (%))

^a^ Serum creatinine concentration > 106 µmol/l

**Table 2:** Patient demographic characteristics, risk factors, and comorbidities of patients with venogenic erectile dysfunction before treatment

Patient characteristics	
Age, years	52 (SD 14)
Nicotine abuse, active	51/253 (20.2%)
Nicotine abuse, former	38/253 (15.0%)
Diabetes mellitus	37/255 (14.5%)
Arterial hypertension	93/254 (36.6%)
Hyperlipidemia	103/253 (40.7%)
Hyperlipoproteinemia a	65/161 (40.4%)
Coronary artery disease	30/253 (11.90%)
Peripheral arterial occlusive disease	18/254 (7.1%)
Cerebrovascular disease	6/253 (2.4%)
Neurological disease(s), nonvascular	20/255 (7.8%)
Prior prostate surgery	11/253 (4.3%)
Chronic prostatitis	7/255 (2.7%)
Renal insufficiency ^a^	19/203 (9.4%)
Alcohol abuse, active	2/255 (0.8%)
Alcohol abuse, former	2/255 (0.8%)
Drug abuse, active	7/254 (2.8%)
Drug abuse, former	5/254 (2.0%)
Hormonal disorder	6/253 (2.4%)
Antiandrogens	1/255 (0.4%)
Antipsychotics	9/255 (3.5%)
Antidepressants	16/255 (6.8%)
Antihistamines	6/255 (2.4%)
Antidiabetics	23/194 (11.9%)
Antihypertensives	89/255 (34.9%)
Aspirin	43/256 (16.8%)
Oral anticoagulation	11/256 (4.3%)
Clopidogrel	5/256 (2.0%)
Statin	49/192 (25.5%)

Mean value (standard deviation (SD)) or number of patients (n/N (%))

^a^ Serum creatinine concentration > 106 µmol/l

**Table 3:** Characteristics of arterial revascularization procedures

Procedural characteristics	
*Number of affected arteries per patient*	
1	263/524 (50.2%)
2	188/524 (35.9%)
3	51/524 (9.7%)
4	21/524 (4.0%)
5	1/524 (0.2%)
*Affected arteries*	
A. penis communis	179/879 (20.4%)
A. pudenda interna, distal	186/879 (21.2%)
A. pudenda interna, proximal	109/879 (12.4%)
A. pudenda interna, middle	234/879 (26.6%)
A. iliaca interna	50/879 (5.7%)
A. cavernosa/A. profunda penis	48/879 (5.5%)
A. glutea inferior	40/879 (4.6%)
A. dorsalis penis	28/879 (3.2%)
A. iliaca communis	5/879 (0.6%)
Contrast material, ml	50 (IQR 40–75)
Balloon angioplasty (PTA)	122/521 (23.4%)
Drug-coated balloon angioplasty	31/521 (6%)
Plain balloon angioplasty	91/521 (17.5%)
*Arterial segments treated with drug eluting stent*	
1 segment	438/524 (83.6%)
2 segments	195/524 (37.2%)
3 segments	50/524 (9.5%)
4 segments	12/524 (2.3%)
5 segments	1/524 (0.2%)
*Technical success*	
1 target artery	503/510 (98.6%)
2 target arteries	224/232 (96.6%)
3 target arteries	59/60 (98.3%)
4 target arteries	18/19 (94.7%)
5 target arteries	1/1 (100%)

The number of patients (n/N (%)) or the median (IQR) is indicated

**Table 4:** Characteristics of venous embolization procedures

Procedural characteristics	
*Access*	
Direct penile puncture	174/188 (92.6%)
Transfemoral	14/188 (7.4%)
*Target vessel*	
Periprostatic venous plexus only	150/188 (79.8%)
Periprostatic venous plexus and V. pudenda interna	25/188 (13.3%)
Periprostatic venous plexus and V. pudenda externa	4/188 (2.1%)
Periprostatic venous plexus and both Vv. Pudenda interna and externa	3/188 (1.6%)
V. pudenda externa	1/188 (0.5%)
V. pudenda interna	2/188 (1.1%)
*Embolization*	
Glubran	101/188 (53.7%)
Histoacryl	73/188 (38.8%)
Magic Glue	14/188 (7.5%)
*Concentration ratio of embolization solution to lipiodol*	
1:1	158/188 (84.0%)
1:2	9/188 (4.8%)
Other	9/188 (4.8%)
Volume of Embolization Solution, ml	2,5 (IQR 2–4)
Additional Coils	26/188 (13.8%)
Contrast agent, ml	8 (IQR 5–20)
Technical success	186/188 (98.9%)
*Phlebography result*	
Stasis	172/188 (91.5%)
Partial stasis	14/188 (7.4%)
No stasis	2/188 (1.1%)

The number of patients (n/N (%)) or the median (IQR) are indicated

**Table 5:** Safety results of patients treated for arterial and/or venous erectile dysfunction

Arterial revascularization	
*Post interventional, 11 days (SD 22)*	
Puncture complications^a^	110/492 (22.4%) ^b^
*Follow-up, median 24 months (IQR 11–48)*	
Surgical procedure required	9/393 (2.3%) ^c^
Repeated arterial intervention performed during follow-up	49/399 (12.3%)
TLR	13/47 (27.7%)
TVR	11/47 (23.4%)
Perineal skin defect	0/432 (0%)
Death	2/437 (0.5%)
Venous embolization	
*Periprocedural*	
Pain between discharge and first postinterventional visit requiring ibuprofen medication	20/188 (10.6%)
Nontarget-vessel embolization	3/188 (1.6%)
*Post interventional, 7 days (SD 5)*	
Puncture complications	3/169 (1.8%) ^a^
*Follow-up, median 3 months (IQR 1–13)*	
Surgical procedure required (anal, penile, perineal)	0/135 (0%)
Perineal skin defect	0/134 (0%)
Repeat embolization performed during follow-up	13/188 (6.9%)
Death	0/135 (0%)
Arterial revascularization plus venous embolization	
*Periprocedural*	
Pain between discharge and first postinterventional visit requiring ibuprofen medication	1/7 (14.3%)
*Post interventional, 7 days (SD 5)*	
Puncture complications ^a^	6/59 (10%) ^d^
*Follow-up, median 21 months (IQR 11–34.5)*	
Surgical procedure required	1/61 (2%) ^c^
Repeated catheter intervention performed	37/61 (61%)
Perineal skin defect	0/61(0%)
Death	0/61 (0%)

The number of patients (n/N (%)) is indicated

^a^ Hematoma occurred in 3 patients, all of whom were successfully managed conservatively

^b^ Hematoma occurred in 107 patients, all of whom were successfully managed conservatively. Additionally, a pseudoaneurysm was present in 3 of those patients

In 2 patients, a pseudoaneurysm occurred without a hematoma, and in one patient, an arteriovenous fistula was present

^c^ No anal, penile, or perineal surgery was performed

^d^ Hematoma occurred in 3 patients, all of whom were successfully managed conservatively

**Table 6:** Clinical improvement by ≥ 4 score points in the IIEF-6 score in patients with arterial and/or venous erectile dysfunction

Score improvement by ≥ 4 Score points ^a^	
After arterial revascularization Follow-up after 30.4 months (SD 25.4) median 24 months (IQR 11–48)	175/402 (43.5%)
After venous embolization Follow-up after 9.2 months (SD 11.9), median 3 months (IQR 1–13)	62/140 (44.3%)
After arterial revascularization plus venous embolization 27.0 (SD 20.7) median 21 months (IQR 11–34.5)	28/54 (52%)

The number of patients (n/N (%)) is indicated

^a^ Corresponds to the minimal clinically relevant improvement in the IIEF-6 score

IIEF-15, International Index of ED (Questions 1–15; max. 75 points)

IIEF-6, Domain questionnaire for erectile function (Questions 1, 2, 3, 4, 5, 15; max. 30 points)

### Results of subgroup “arterial revascularization”

Patients’ demographic characteristics, risk factors, and comorbidities of patients with arteriogenic erectile dysfunction are demonstrated in Table 1. Characteristics of arterial revascularization procedures are demonstrated in Table 3. Technical success was achieved in 94.7% to 98.6% of the patients. Safety results of patients treated with arterial revascularization are summarized in Table 5. Of note, 2 out of 437 patients (0.5%) died during follow-up approximately 2 years postintervention, but with no causal link to the procedure.

Clinical improvement in the IIEF-6 score of ≥ 4 points was found in 175/402 (43.5%) of patients 30 months after arterial revascularization (Table 6, Fig. 4). According to PGI-I, patients reported improved erectile function after arterial revascularization in 61% (226/369), and 244 out of 362 (67.4%) patients stated that they would undergo the procedure again (Fig. 7).

In a univariate analysis, none of the following factors were identified as risk factors for a lack of improvement in IIEF-6 ≥ 4 points after arterial revascularization: type II diabetes mellitus (OR 0.78, 95% CI: 0.47–1.30; *p* = 0.27); a history of prostatectomy (OR 0.97, 95% CI: 0.43–2.63; *p* = 0.89); and age ≥ 65 years (OR 1.03, 95% CI: 0.68–1.56; *p* = 0.89).

### Results of subgroup “venous embolization”

Patients’ demographic characteristics, risk factors, and comorbidities of patients with venogenic erectile dysfunction are demonstrated in Table 2. Characteristics of venous embolization procedures are demonstrated in Table 4. Technical success was achieved in 186/188 (98.9%) of patients. There were no major complications or periprocedural deaths following venous embolization procedures. Safety results of patients treated with venous embolization are summarized in Table 5. Notably, three patients (1.6%) experienced nontarget embolization (pulmonary embolisms due to fragments of the embolic agents) during venous leak embolization, which required a 3-month course of oral anticoagulation, but without long-term consequences (CIRSE grade 3 complication).

Nine months after venous leak embolization, an IIEF-6 score improvement of ≥ 4 points was observed in 62/140 (44.3%) of patients (Table 6, Fig. 5). According to PGI-I, patients reported improved erectile function after venous embolization in 72% (102/142), and 107 out of 136 (78.7%) patients stated that they would undergo the procedure again (Fig. 8).

The following factors were not predictive of a lack of improvement following venous embolization: type II diabetes mellitus (OR 1.15, 95% CI: 0.45–2.97; *p* = 0.77); prostatectomy (OR 1.04, 95% CI: 0.22–4.81; *p* = 0.96); and age ≥ 65 years (OR 1.67, 95% CI: 0.75–3.70; *p* = 0.21).

### Results of subgroup “arterial revascularization plus venous embolization”

Safety results are summarized in Table 5. There were no major complications or periprocedural deaths. Clinical improvement in the IIEF-6 score of ≥ 4 points was found in 28/54 (52%) of patients after they underwent both arterial and venous interventions (Table 6, Fig. 6). According to PGI-I, patients reported improved erectile function after both arterial revascularization and venous embolization in 68% (38/56), and 49 out of 56 (87.5%) patients stated that they would undergo the procedure again (Fig. 9).

## Discussion

ED is a global disease burden, with an estimated global patient incidence of 322 million in 2025 [11]. Among patients with ED, approximately 30%–50% do not respond sufficiently to PDE5is, or they have side effects making them potential candidates for endovascular therapy. Therefore, the potential target patient population for endovascular therapy is considerably larger than that for peripheral arterial disease [22, 23].

Endovascular therapy for arterial and venous ED has emerged in the past decade. Although not yet widely addressed by vascular interventionalists in many parts of the world, the diagnosis and endovascular treatment of vascular ED are fascinating and challenging.

ED clearly requires a multidisciplinary approach that is based on current scientific evidence, and patients should be referred for endovascular therapy only if vasoactive drugs are not effective and if urological causes of ED are excluded.

ED is an early marker of cardiovascular disease and an independent risk factor for future cardiovascular events, allowing early diagnosis and secondary prevention of atherosclerosis. Early recognition of atherosclerotic disease in ED patients and subsequent medical treatment of cardiovascular risk factors was reported to be associated with a reduced incidence of cardiovascular major adverse events and was reported to be cost effective [24].

Since 2016, we have gained invaluable experience by performing more than 1000 catheter-based therapies for the treatment of severe vasculogenic ED.

### Vascular diagnosis and management of combined arteriovenous ED

Duplex sonography facilitated by intracavernous prostaglandins is an important diagnostic tool in vascular ED [12, 23]. Patients with severe ED that do not sufficiently respond to vasoactive agents such as PDE-5is or intracavernous alprostadil are very likely to have a vascular etiology. A combination of mixed arteriogenic and venogenic changes was reported to be present in more than 40% of ED patients [25–27].

Pharmacological penile duplex sonography has several limitations, particularly when diagnosing venous leak. Continuous venous outflow can falsely lower the peak systolic velocity (PSV), mimicking arterial insufficiency and leading to misclassification [12]. Moreover, fixed end systolic velocity (EDV) thresholds (e.g., > 5 cm/s) often fail to reflect individual hemodynamic variability and may have limited specificity, especially in mixed vascular pathologies [23]. These diagnostic challenges underscore the need for dynamic assessment strategies. One promising approach is ultrasound-guided venous compression. By combining high-resolution duplex sonography with real-time mechanical modulation of venous outflow, this method enables functional restoration of physiological EDVs and reveals characteristic PSV shifts. As demonstrated in recent studied, this method matched the diagnostic sensitivity of CT cavernosography while remaining noninvasive [23, 25]. In addition, in patients with mixed arteriogenic and venogenic ED, the individual impact of each arterial and venous pathology on ED symptoms may not be quantifiable prior to endovascular treatment.

Over the years, our approach in patients with mixed arteriovenous disorders has matured to stipulate primary arterial revascularization followed by venous embolization if clinically required (i.e., in patients without a sufficient erectile response to arterial revascularization alone, despite the use of maximum doses of PDE5is) [10]. This approach is based upon the following considerations: First, we empirically had greater interventional experience with arterial revascularization than with venous embolization. Second, adequate arterial supply may be necessary to correctly assess the presence of a venous leak. Third, we observed that the use of a radiopaque liquid embolic agent for venous leak embolization may impair angiographic visualization in the case that arterial revascularization is required subsequent to venous leak embolization.

### Clinical outcomes

The primary aim of endovascular therapy in patients with severe ED is to reestablish erections sufficient for satisfactory sexual intercourse. Our results confirm the findings of smaller series summarized in a recent meta-analysis and demonstrate that outcomes may remain stable in the long term [3]. Endovascular treatment of ED was demonstrated to be safe, with risks comparable to those of other endovascular procedures, such as percutaneous transluminal angioplasty for peripheral arterial disease (PAD), prostate artery embolization, or endovenous laser therapy for varicose veins.

Given that the SwissPower registry did not exclude patients with comorbidities potentially affecting erectile function, such as diabetes mellitus and prostatectomy, and that we included only men with severe ED who did not sufficiently respond to vasoactive agents, for whom a penile prosthesis would have been the only, much more invasive alternative, we interpret our proceedings and results as favorable.

Our findings on the treatment of mixed arteriovenous pathologies indicate further potential for clinical development [10]. Thus, if clinical improvement after technically successful arterial revascularization is missing, detailed scrutiny of veno-occlusive function and potential embolization of venous leak should be considered.

In the future, registry data that includes greater numbers of patients should be used to define the impact of risk factors such as diabetes mellitus, prostatectomy, and the use of medications such as antidepressants for unfavorable outcomes.

### Technical considerations in arterial revascularization for ED

Compared with external iliac arteries, erection-related arteries are considerably smaller in diameter, making them more prone to elastic recoil and acute thrombotic reocclusion [28]. At present, the optimal arterial revascularization strategy for erection-related arteries remains to be defined. Given the extent of arterial recoil after plain balloon angioplasty alone and considering that the credibility of catheter-based therapies needs to be established, we adopted a primary drug-eluting stent policy early on [7]. The use of low-profile Sirolimus-eluting stents was associated with restenosis rates as low as 15.4% at 9 months despite the relatively small diameters of the treated arteries [29].

However, considering the low profile of erection-related arteries and the relatively young age compared with average peripheral arterial disease patients, a leave-nothing-behind strategy may be favorable for arterial ED patients [30]. Thus, the role of drug-coated balloons and absorbable stents requires future scrutiny.

### Technical considerations in venous leak embolization for ED

If venous leak is suspected on the basis of penile duplex sonography, CT cavernosography should be performed to verify the presence of venous leak and to acquire detailed, site-specific information regarding its classification for treatment planning [10, 15].

Treatment of venous leak is performed using various methods, such as surgical ligation, sclerotherapy, and embolization. Embolization therapy is minimally invasive when either anterograde access via a deep dorsal penile vein or retrograde access via a femoral vein performed in an angio suite is used [9, 11]. On the other hand, surgical treatment, with or without embolization, is more invasive and typically requires an operating room and general anesthesia [31]. Furthermore, the success rates of surgical ligation of the deep dorsal vein and its collaterals for spontaneous erection were reported to be no more than 25%, whereas venous leak embolization demonstrated a clinical success rate of nearly 60% up to 20 months posttreatment, and an improvement in erectile function was reported in more than 70% of patients [11, 32–35].

The objective of embolization therapy is to achieve adequate embolization of the efferent pelvic veins, including the periprostatic, internal, and external pudendal veins. Since local anesthesia is rendered difficult, periinterventional sedoanalgesia is performed. Anterograde access via a deep dorsal penile vein is preferred, and the use of this access route has been reported to be successful in the majority of cases (86%) [11].

However, ultrasound-guided percutaneous deep dorsal vein puncture has a learning curve and may be technically challenging owing to penile mobility, a rough penile fascia (Buck’s fascia), a small venous caliber or venous spasm [16]. Subsequent to venous access, a stiff 3-F inner dilator is inserted to penetrate Buck’s fascia into a deep dorsal penile vein [17]. In the majority of cases, this introducer can be advanced and positioned with its tip in the center of the venous leak near the radix penis. If this fails, a microcatheter should be used. Prior to embolization, a venogram is recommended to ensure correct positioning of the catheter tip in the venous leak center. In cases where not all draining veins are included, selective microcatheterization of these veins may be necessary. Protective distal coil embolization, as performed in 13.8% of venous leak embolization cases in the present study, is conducted in veins exhibiting rapid outflow on venogram before the application of liquid glue, with the aim of preventing its migration to the femoral or iliohypogastric veins. Therefore, coils are oversized by up to 50% compared with the target vein diameter to mitigate the risk of coil migration. On the other hand, retrograde catheterization of internal and external pudendal veins through transfemoral access may present additional complexities [18]. Using this retrograde approach, it may be challenging to catheterize a venous leak because of the limited ability to visualize the target vein with contrast material.

For embolization, slow and steady controlled administration of a liquid embolic agent should consistently be conducted with the patient performing the Valsalva maneuver in addition to potential distal coil embolization of draining veins to prevent nontarget embolization, particularly pulmonary embolism. A non-target embolization with glue to the pulmonary arteries may cause mechanical vascular obstruction, acute pulmonary hypertension, right ventricular failure, severe hypoxia, and potentially sudden cardiovascular collapse.

We have consistently used a 1:1 mix of glue and lipiodol, while the amount of liquid embolic agent injected has increased from approximately 3 ml at the beginning of our experience to approximately 6 ml to date.

Persistence or recurrence of venous leak postembolization may occur in approximately 10% of cases [11]. Recurrence may occur because of the re-establishment of blood flow in veins that have been previously treated or the formation of new collateral veins. Collateral veins may develop as a compensatory response to redirected blood flow resulting from venous insufficiency, indicating persistent venous dysfunction. Persistence was observed when a target vein was inadequately embolized because of insufficient opacification and/or suboptimal positioning of the catheter tip.

### Limitations

Although the study design was prospective, it lacked a control group, and the potential for a placebo effect cannot be fully discounted. Some study patients were lost to follow-up because they may have changed their contact information, making them difficult to reach. Others lose interest or motivation to continue participating once endovascular treatment is performed. Time constraints may further limit continued participation. Furthermore, patients were administered 5 mg tadalafil daily for 3 weeks following endovascular treatment. Notably, all the included patients were unresponsive to maximum doses of PDE5is, and 90% of the patients were nonresponders to intracavernous alprostadil at baseline. Consequently, this approach does not allow us to ascertain the impact of endovascular treatment without the use of PDE5is.

## Conclusion

Endovascular therapy for severe vascular ED is a rapidly unfolding treatment option with a safety profile comparable to that of similar interventions for PAD or varicose veins. These often challenging interventions are reserved for experienced interventionalists with skills in the revascularization of small-caliber arteries and embolization therapy. Further studies should investigate ideal antirestenosis concepts in arterial intervention and help to better define subgroups of patients with no or suboptimal response.

## Data Availability

Study data and materials are available.

## Abbreviations

ED: Erectile dysfunction
IIEF: International Index of Erectile Function
PGI-I: Patient Global Impression of Improvement
PDE5is: Phosphodiesterase-5 inhibitors
MCID: Minimum clinically important difference
PSV: Peak systolic velocity
EDV: End systolic velocity
CT: Computed tomography
PAD: Peripheral arterial disease
PTA: Percutaneous transluminal angioplasty
SD: Standard deviation
IQR: Interquartile range
