# Correction: Endovascular therapy for erectile dysfunction: insights from more than 1000 treatments

**DOI:** 10.1186/s42155-026-00723-6

**Published:** 2026-07-03

**Authors:** Nicolas Diehm, Patrick Duy Dang Do, Madlen Röllig, Dominik Müller, Sebastian Sixt, Michael Glenck, Andreas Gutwein, Tamara Walser, Chiara Brechbühl, Martin Christian Schumacher, Dai-Do Do, Hanno Hoppe

**Affiliations:** 1 Vascular Institute Central Switzerland, Aarenaustrasse 2B, Aarau, 5000 Switzerland; 2 Faculty of Medicine, University of Bern, Murtenstrasse 11, Bern, 3008 Switzerland; 3 University of Applied Sciences Furtwangen, Jakob-Kienzle-Strasse 17, Villingen-Schwenningen, 78054 Germany; 4 Department of Vascular Surgery, Inselspital University Hospital Bern, Freiburgstrasse 20, Bern, 3008 Switzerland; 5 Vascular Center Biel, Bahnhofstrasse 14, Biel, 2502 Switzerland; 6 Microtherapy Center Bern, Lindenhofgruppe, Bremgartenstrasse 117, Bern, 3012 Switzerland; 7 Hirslanden Clinic Aarau, Urology Center, Aarau, Schänisweg 5001 Switzerland; 8 Campus Stiftung Lindenhof Bern, Swiss Institute for Translational and Entrepreneurial Medicine, Hochfeldstrasse 41, Bern, 3012 Switzerland; 9 Department of Diagnostic, Interventional and Pediatric Radiology (DIPR), Inselspital, Bern University Hospital, University of Bern, Rosenbühlgasse 27, Bern, 3010 Switzerland; 10 Department of Health Sciences and Medicine, University of Lucerne, Frohburgstrasse 3, Postfach 4466, Lucerne, 6002 Switzerland; 11 Department of Angiology, Inselspital University Hospital Bern, Freiburgstrasse 20, Bern, 3008 Switzerland

**Correction: CVIR Endovasc 9, 23 (2026)**

**https://doi.org/10.1186/s42155-026-00666-y**

Following the publication of the original article [1], the authors reported the affiliation of the first author Nicolas Diehm is incomplete. This would ultimately result in refusal of the University of Bern to acknowledge this piece of work for their thesis student, Patrick Day Dang Do.

May the authors therefore kindly ask to add the following affiliation for first author Nicolas Diehm: “Department of Angiology, Inselspital University Hospital Bern, Freiburgstrasse 20, Bern, 3008, Switzerland”
